# Grinding exfoliation for scalable production of 2D materials

**DOI:** 10.1093/nsr/nwz202

**Published:** 2019-12-13

**Authors:** Kostya S Novoselov, Qi Ge, Daria V Andreeva

**Affiliations:** 1 Department of Materials Science and Engineering, National University of Singapore, Singapore; 2 National Graphene Institute, University of Manchester, UK; 3 Chongqing 2D Materials Institute, China

Over the past few years the utilisation of graphene in various areas of industry has strongly intensified [1]. Applications in thermal management, composite materials and energy consume an increasing amount of this two-dimensional (2D) material in the form of powder or colloidal suspensions [2]. Different applications, however, require different forms of this 2D crystal. Thus, composite materials would benefit from large (a few microns), one to a few layers thick, moderately functionalised crystals to facilitate the most efficient stress transfer, and energy applications (batteries) would require slightly functionalised monolayer flakes with a broad distribution of sizes in order to create a fractal conducting network.

Even though considerable efforts have been spent on the development of different methods of the production of graphene flakes [3–5], industry still requires a fast, low-cost technology that produces flakes of 2D materials of a guaranteed quality. All existing processes are either expensive and cumbersome (like the graphene oxide way, where significant amounts of chemicals need to be spent on oxidation, exfoliation and reduction of graphene) or give a very low yield combined with small sizes of the flakes (sonication or microfluidics). Significant progress has been achieved with electrochemical exfoliation. However, this process only works with conductive 2D materials.

Thus, industry would significantly benefit from a new, low-cost way of producing high quality flakes of 2D crystals that would work equally well on conductive and insulating materials. In a recent report [6] by the research group headed by Hui-Ming Cheng and Bilu Liu from the Shenzhen Geim Graphene Centre, the researchers described a very simple and elegant way to enhance the shear forces on 2D materials for efficient exfoliation.

The use of shear forces (when the layers in a layered crystal are forced to slide with respect to each other) is the most energy efficient and productive way of exfoliation: with sliding, the flakes do not break, and thus the result, on average, is larger flakes. Several other techniques (shear mixing [5], microfluidic exfoliation [4]) rely exactly on such sliding for the production of 2D inks. The issue is, however, how to apply such shear forces efficiently. Shear mixing has a rather low yield because the number of particles that are in contact with the blades (where shear forces are applied due to finite viscosity of the media) is small. In microfluidic technology the large sliding forces are achieved very efficiently inside of the microchannel – again, acting only on a small volume of the suspension at any given moment of time.

The group from the Shenzhen Geim Graphene Centre found a way of applying large sliding forces to a large volume of layered crystals at the same time. They mixed the powder of 2D materials with abrasive material (silicon carbide) with particles of roughly similar size. The mixture was compressed in a rotating press. Without abrasive particles the 2D crystals would simply compact flat and the fraction of the applied force transferred to the shear force between layers would be limited by the friction coefficient between individual flakes, which is usually very low for 2D materials. Instead, abrasive particles create irregularities in the structure of the compacted film, which produces a very efficient way of transferring the applied pressure to the shear forces.

Because the shear forces are now being applied to all flakes simultaneously, an extremely high production rate of 0.3 g/h (with a yield of 50%) is possible, which is 10 times better than the next most efficient process of ball milling. The average size for the 2D layers was above 1 μm and the average thickness was ∼4 nm.

The announced figures should allow an increase in the production of the 2D crystals beyond graphene and this will open further applications for such materials in applications such as functional composites, energy, printable electronics, etc.

***Conflict of interest statement.*** None declared.
